# Daily enteral feeding practice on the ICU: attainment of goals and interfering factors

**DOI:** 10.1186/cc3504

**Published:** 2005-03-22

**Authors:** JM Binnekade, R Tepaske, P Bruynzeel, EMH Mathus-Vliegen, RJ de Hann

**Affiliations:** 1 Research Nurse, Department of Intensive Care, Academic Medical Center, University of Amsterdam, Amsterdam, The Netherlands; 2 Intensivist, Department of Intensive Care, Academic Medical Center, University of Amsterdam, Amsterdam, The Netherlands; 3 Dietician, Department of Dietetics, Academic Medical Center, University of Amsterdam, Amsterdam, The Netherlands; 4 Gastroenterologist, Department of Gastroenterology, Academic Medical Center, University of Amsterdam, Amsterdam, The Netherlands; 5 Clinical Epidemiologist, Department of Clinical Epidemiology and Biostatistics, Academic Medical Center, University of Amsterdam, Amsterdam, The Netherlands

## Abstract

**Background:**

The purpose of this study was to evaluate the daily feeding practice of enterally fed patients in an intensive care unit (ICU) and to study the impact of preset factors in reaching predefined optimal nutritional goals.

**Methods:**

The feeding practice of all ICU patients receiving enteral nutrition for at least 48 hours was recorded during a 1-year period. Actual intake was expressed as the percentage of the prescribed volume of formula (a success is defined as 90% or more). Prescribed volume (optimal intake) was guided by protocol but adjusted to individual patient conditions by the intensivist. The potential barriers to the success of feeding were assessed by multivariate analysis.

**Results:**

Four-hundred-and-three eligible patients had a total of 3,526 records of feeding days. The desired intake was successful in 52% (1,842 of 3,526) of feeding days. The percentage of successful feeding days increased from 39% (124 of 316) on day 1 to 51% (112 of 218) on day 5. Average ideal protein intake was 54% (95% confidence interval (CI) 52 to 55), energy intake was 66% (95% CI 65 to 68) and volume 75% (95% CI 74 to 76). Factors impeding successful nutrition were the use of the feeding tube to deliver contrast, the need for prokinetic drugs, a high Therapeutic Intervention Score System category and elective admissions.

**Conclusion:**

The records revealed an unsatisfactory feeding process. A better use of relative successful volume intake, namely increasing the energy and protein density, could enhance the nutritional yield. Factors such as an improper use of tubes and feeding intolerance were related to failure. Meticulous recording of intake and interfering factors helps to uncover inadequacies in ICU feeding practice.

## Introduction

Protein energy malnutrition is a major problem in severely ill hypercatabolic patients in the intensive care unit (ICU) [1]. Early initiation of enteral nutrition has proved to be beneficial, with significant positive effects on septic complications, and has been shown to improve the outcome when compared with parenteral nutrition. Enteral nutrition guarantees the preservation of gut mass and prevents increased gut permeability to bacteria and toxins [2-5]. In addition, the gut-associated lymphoid tissue is better maintained [4].

Over the years, enteral nutrition has improved with regard to techniques, materials and composition, and has gained popularity because of its lower cost and lower rate of complications compared with parenteral nutrition. This is also reflected in our intensive care by an increased use of enteral nutrition from 16.7% of total patient days in 1992 to 53.8% in 2001, and a slightly decreased use of parenteral nutrition, from 19% of total patient days in 1992 to 14% in 2001.

Although this large increase in enteral feeding days has to be considered a step forward, these figures do not show the actual intake of energy and nutrients per patient; that is, the adequacy of feeding. Despite the attention given to the practice of enteral nutrition in daily rounds by intensivists and ICU nurses, we were not adequately and accurately informed as to the adequacy of our feeding practice [6]. Confronted with a growing number of enterally fed patients we decided to develop a daily record, aimed at obtaining a continuous and long-term overall insight into the volume, energy content and amount of proteins administered to and actually received by the patient. The objective of this study was to evaluate the success of enteral nutrition in our ICU and to report the influence of factors presumed to interfere and, being part of the record, to achieve an optimal nutritional intake.

## Materials and methods

### Setting

The study was conducted in a 30-bed intensive care unit with access to patients of all specialties at the Academic Medical Center in Amsterdam, a tertiary care university teaching hospital with 1,000 beds.

### Feeding process

Standard feeding practice involved the continuous administration of enteral feeding solutions over 24 hours. Although a standard feeding protocol was in use (see Additional file 1) the flow rate was often adjusted according to the understanding of the intensivist.

Patients started feeding at 500 ml per day with a build-up of 500 ml per 24 hours until the individually determined intake in terms of volume, proteins and calories was reached. Given an uneventful course a patient would achieve an intake of 2,000 ml within 5 days. However, to compensate for interruptions of feeding, the intake was targeted at a 20% higher volume. The optimal feeding target of 2,000 kcal per 24 hours therefore became 2,400 kcal per 24 hours after adjustment.

### Data collection

Patients admitted to the ICU and receiving enteral nutrition for at least 48 hours were eligible. The study duration for each patient was limited to 30 days. In this retrospective database study we extracted the daily records of enterally fed patients over a period of 1 year. Records containing a single oral-nutrition or total parenteral-nutrition day, or records that lacked a prescription of desired intake, were excluded from the analysis.

Feeding factors assumed to interfere with enteral nutrition and noted in the record were as follows: first, the type of feeding tube (gastric tube, duodenal tube, percutaneous endoscopic gastrostomy, or needle catheter jejunostomy (NCJ)); second, the type of formula with different energy content (100 to 204 kcal/100 ml) and protein content (4 to 7 g/100 ml) and normal or predigested semi-elemental form; third, gastric retention; fourth, therapeutic interventions (mechanical ventilation, endotracheal tube *in situ*, extubation/intubation, spontaneous respiration, tracheostomy, continuous veno-venous haemofiltration, prone position, and preparation for computed tomography scan); and fifth, medication (lactulose, cisapride, midazolam–morphine, morphine, propofol, vasopressors, inotropics and pantoprazol).

The feeding record was coupled to other databases to extract data on gender, age, length of stay and referral specialty in the ICU, the Acute Physiology and Chronic Health Evaluation score (APACHE II) and the therapy intensity with the Therapeutic Intervention Score System (TISS). The TISS scores were calculated for each patient and subdivided into four categories, classifying the patient's need for ICU care: in category 1 the score was less than 10 points (no need for ICU care); 2, a score of 10 or more to less than 20 points (physiologically stable condition with prophylactic overnight observation); 3 a score of more than 20 to less than 40 points (physiologically stable but requiring intensive nursing and monitoring); and 4, a score of more than 40 points (unstable condition requiring intensive physician and nursing care) [7]. The APACHE II [8] was scored upon admission (within 24 hours); APACHE II scores could range from 0 to 71, with higher scores indicating a more severe illness.

### Reliability of the record

As the record had to be filled in by several staff members, its reliability had to be tested. An interobserver study was performed between two regular keepers of the record (a dietician and an intensivist). Nursing charts of 42 feeding days for 14 patients (3 days per patient) were evaluated by three different observers and the data were entered into the record.

### Analyses

Descriptive statistics were used to characterize patients. Successful intake was defined as a patient's receiving 90% or more of the prescribed amount of feeding. The difference between the prescribed amount and the tube feeding actually administered was expressed as a percentage and its associated 95% confidence interval (95% CI). The percentages (95% CIs) of realized energy and protein needs were based on a ideal 30 kcal per kg of body weight [9] and 1.5 g of protein per kg of body weight [10,11], respectively. The volume, energy and protein intake were stratified by type of formula and arranged by type of enteral route (total of 28 strata). Patients with zero intake but having a feeding prescription remained in the analysis.

Univariate analysis was performed to assess determinants of successful intake with regard to patients and feeding factors. Before inclusion into the model the independence of these explanatory variables had to be determined. The most common value of the categories (referral specialty, type of feeding tube and type of formula) were used as reference category (odds ratio of 1). Each category of the predictor variable was then compared with the reference category for categorical variables.

Significant variables in the univariate analysis (*P *≤ 0.10) from patients and feeding factors were forced into the multivariate logistic regression model (enter method).

The results of the univariate analysis were also compared for the data set of the complete feeding period and a data set of the first three feeding days. Significant differences might show influences of a skewed duration of feeding.

Statistical uncertainty was expressed as 95% CI. Data were analyzed in SPSS version 11.5.

## Results

In 2001, 1,479 patients were admitted to the ICU. After the removal of elective admissions with a limited stay of less than 48 hours, the crude data set contained 5,859 feeding days. Because the analysis was limited to 30 days of ICU stay, 5,017 days remained. The removal of feeding days with one single day of oral or total parenteral nutrition and the removal of patients who did not receive a prescription for enteral feeding resulted in 3,526 days to be analyzed in 403 patients.

There was a significant difference between neurosurgery and the other specialties for length of ICU stay. APACHE II scores did not differ between medical and neurosurgery patients. Medical patients had the highest APACHE II score, significantly higher than those of surgical and cardiac surgical patients (Table 1).

### Reliability of the record

The test of reliability showed an intra-class correlation (two-way random model) of 0.98 (95% CI 0.96 to 0.99).

### Success of enteral nutrition

During the build-up phase of feeding, the number of successful feeding days increased from 39% (124 of 316) on day 1 to 51% (112 of 218) on day 5. At discharge from the ICU only 4% (14 of 371) of patients received 100 ml/hour or more enteral nutrition. Twenty-five percent (93 of 371) of patients left the ICU with an intake of 80 ml/hour, whereas 71% (264 of 371) of patients received 60 ml/hour or less. Thirty-three patients stayed for longer than 30 days in the ICU; food intake on discharge was therefore not analyzed.

The percentage of successful intake and ideal energy and ideal protein calculated for each type of formula and for each type of enteral route showed an overall picture of deficiency. Of the 28 strata, 21 were analyzable (Table 2). Ten strata showed the highest percentage for volume, another ten for energy and only one for protein. In eight strata protein turned out to be less important than volume, whereas in seven strata protein was less important than the percentage of energy (Table 2).

### Factors interfering with successful administration of enteral feed

#### Tube location

The percentage of days with successful feeding was smallest for gastric tubes and greatest for duodenal/jejunal tubes (Table 3). The NCJ had significantly more successful feeding days than the duodenal tube; the difference was 19% (95% CI 27 to 10) (Table 3).

#### Gastric retention

Patients fed by duodenal tube had the highest gastric retention, with a mean of 558 (95% CI 523 to 593) ml/24 hours. The mean gastric retention among patients with a gastric tube was 159 (148 to 170) ml/24 hours. Of these, a mean of 121 (110 to 132) ml gastric retention over a 24-hour period was discarded by the nurse instead of being given back to the patient. On the assumption that the gastric retention in patients with a gastric tube contained mainly tube feeding, the amount of nutrition delivered would decline to a mean of 1,066 (1,034 to 1,097) ml/24 hours. In this scenario, the removal of gastric retention fluids caused a decline in the percentage of successful feeding of 6% (95% CI 4 to 10%) to a 42% (1,024 of 2,455) success rate. Although the protocol dictated that gastric retention volumes of less than 200 ml in 6 hours had to be given back, 34% (266 of 791) of gastric retention volumes of less than 200 ml/hour were discarded.

#### TISS scores

Category 3 TISS scores were present on 66% (2,349 of 3,577) and category 4 TISS scores on 31% (1,106 of 3,577) of patient days. Among category 3 TISS patients the success rate of feeding (at least 90% intake) was 55% (1,285 of 2,349) in comparison with a 45% (498 of 1,106) success rate of feeding among category 4 patient days; this is a difference of 10% (95% CI 6 to 13%).

### Multivariate analysis

Because of significant collinearity between mechanical ventilation and other variables, such as endotracheal tube, extubation, intubation, spontaneous respiration and tracheostomy, only mechanical ventilation was included in the analysis.

A comparison of the results of the univariate analysis between the complete data set and a subset of the first three feeding days did not reveal any important differences.

Univariate analysis of 32 potential determinants of successful intake revealed 12 significant variables (*P *≤ 0.10; not presented). The subsequent multivariate logistics regression analysis resulted in 11 significant variables (*P *≤ 0.05) (Table 4).

Both the NCJ and semi-elemental formula showed the odds ratios as to successful feeding, 3.32 and 3.02, respectively, both to be interpreted against the reference, i.e. the gastric tube and standard feeding formula (Table 4). In addition, a gastric retention of less than 200 ml and a length of stay above the median was related to improved success of feeding. Of the remaining interventions, the administration of contrast via the tube, the need for prokinetic drugs, TISS and elective admission showed an adverse effect on the success of feeding (Table 4).

## Discussion

With the use of a meticulous, daily record of the ICU feeding practice we evaluated the feasibility of prescribed enteral feeding for a 1-year period. The prescribed nutritional volume turns out to be hardly feasible in the patients involved in our study. When actual intake is compared with ideal energy and protein needs, protein shows the largest overall deficit. Current feeding practice (including the 5-day build-up schedule for enteral nutrition) fails to provide ICU patients with adequate nutrition.

Other studies found comparably bad results. A prospective cohort study among 99 ICU patients found that only the half of patients achieved tolerance of the feeding regime (90% of estimated energy for more than 48 hours) [12].

Better results were found in a multicenter prospective study that followed 193 patients during 1,929 patient days. An average of 76% of the prescribed feed was delivered to the patient. They also concluded that using well-defined protocols significantly improved the intake [13].

A prospective study in ICUs and coronary care units revealed that barely one-half of the 44 patients studied met their caloric requirements because of underordering by physicians and reduced delivery arising from frequent and inappropriate cessation of feeding [14].

Another prospective study found also a low caloric intake in 51 enterally fed ICU patients for whom 78% of the mean caloric amount required was prescribed and 71% was actually delivered [15].

An audit of 40 ICU patients for which the ideal feeding target was calculated by the Harris–Benedict equation. Patients received only 51% of these energy requirements during the 7-day study period [16].

A cross-sectional survey of 66 responding dieticians of ICUs revealed that among patients receiving enteral nutrition only 58% met their prescribed energy and protein needs [17].

Although we were aware of these studies, we did not expect this result until we kept these records. Despite having at our disposal an enteral feeding protocol and despite daily bedside consultations with the intensivist, nurse and dietician, only 50% of the enterally fed patients achieved a successful intake at the end of a 5-day feeding build-up scheme. Although a further improvement in intake occurred as the ICU stay was prolonged, the overall success per feeding day remained low during the ICU stay. Apparently, implementation of a protocol, once it has been set out and accepted, is difficult and needs more attention [13,18,19].

The feeding with a NCJ resulted in odds ratios that favor this enteral route over the gastric tube. In addition semi-elemental formula seemed to be three times better than standard formula (Table 4). In part, this might have been confounded by the use of either duodenal tubes or NCJ, because the NCJ showed the fewest problems in use. Because of this and because it concerned a small group of patients, we cannot unambiguously recommend semi-elemental formula although others have done so [20,21].

Disordered upper gastrointestinal tract motility frequently occurs in ICU patients [22], yet the gastric tube remains the first and simplest choice and the easiest way of starting enteral nutrition. This does not detract from the significant number of patients who have to be switched to a duodenal tube because of persisting gastric retention. We also found that nurses tended to overestimate gastric retention as a risk factor and, more importantly, violated the protocol by discarding a gastric retention volume of less than 200 ml over 6 hours. This behavior might be the result of a misplaced ambition to achieve safer care. Although the measurement of gastric retention is an important tool for guaranteeing safe enteral feeding, no difference is reported between gastric tube and duodenal tube use among ICU patients in terms of aspiration and nosocomial pneumonia. Moreover, the insignificant role of gastric retention levels of up to 250 ml has been reported [23-26].

Using the feeding tube to administer contrast for a CT scan precludes the use of the tube for administering nutrition. In general, a high therapy intensity reflected by a high TISS score indicated a more difficult feeding practice because the subject was more critically ill. This might also reflect the lower priority given in the care routine for optimal continuation of the feeding process in comparison with the efforts taken to support patients in need of ventilation and assisted circulation.

Improvement of nutritional intake can be achieved by implementing simple rules, such as limiting the interruption of enteral nutrition because of diagnostic or therapeutic interventions, a quick replacement of accidentally removed tubes, and giving back gastric retention of less than 250 ml [14,27,28].

Whereas a high TISS score did seem to interfere with the administration of enteral nutrition, the severity of illness did not. It took several days for 50% of the patients to achieve an optimal intake, which to some extent might reflect the unstable physical condition of the ICU patient. This is also shown by the relationship between success of feeding and prolonged ICU stay.

A limitation of this study is that we did not collect or analyze a nutritional anamnesis or patient outcome data. We have focused on measurable aspects of feeding practice. It will be worthwhile to expand the continuous recording to include a (nutritional) anamnesis of the patient. Improving the information load of this record would also require more information about outcome.

## Conclusion

Evaluation of feeding practices has revealed otherwise unnoticed, yet disappointing, results. Although the recording process in itself does not improve feeding practice it might lead to the recognition that the patient is underfed while being fed and that ways have to be found to improve feeding practice, namely by implementing protocols for feeding and gastric retention measurements.

## Key messages

• 	A long-term recording of the ICU nutritional intake revealed an unsatisfactory enteral feeding process.

• 	Factors such as an improper use of tubes and feeding intolerance related to failure of nutritional intake.

• 	Better use of relative successful volume intake by increasing energy and protein density could enhance the nutritional yield.

## Abbreviations

APACHE = Acute Physiology and Chronic Health Evaluation; CI = confidence interval; CT = computed tomography; ICU = intensive care unit; NCJ = needle catheter jejunostomy; TISS = Therapeutic Intervention Score System.

## Competing interests

The author(s) declare that they have no competing interests.

## Authors' contributions

JMB built the database, analyzed the data and wrote the article. RT performed the data collection, performed the interobserver study and co-wrote the article. PB performed data collection and participated in the interobserver study. EMHMV supervised the writing of the article and co-wrote the article. RJH supervised the statistical analysis and the final draft of the article. All authors read and approved the final manuscript.

## Supplementary Material

Additional File 1 A pdf file containing a table listing the protocol outline. Click here for file

## Figures and Tables

**Table 1:** Characteristics of ICU patients recorded in the 1-year enteral feeding practice (*n *= 404)

Characteristic	Value
Male patients, %	63 (256 of 404)
Age, years (mean ± SD)	60 ± 17
Length of stay, days (median and IQ)	
Overall	8 (5/16)
Medical (*n *= 112)	9 (6/18)
Surgical (*n *= 117)	8 (5/16)
Cardiac surgery (*n *= 122)	7 (5/14)
Neurosurgery (*n *= 53)	10 (6/16)
APACHE II score (mean ± SD)	
Overall	18 ±7
Medical	23 ± 7
Surgical	17 ± 6
Cardiac surgery	16 ± 5
Neuro(surgery)	20 ± 6

IQ, interquartile range.

**Table 2:** Percentage of volume of formula actually delivered (intake/prescribed)

Type of formula (kcal/protein)	Enteral route	*n*	Successful feeding days, % (95% CI)		
			
			Volume	Energy	Protein
Standard (100/4)	Gastric tube	1,309	66 (64–68)	47 (45–50)	37 (35–38)
(*n *= 1,760)	PEG	11	87 (67–107)	86 (44–128)	51 (33–70)
	Duodenal tube	392	81 (79–84)	71 (66–75)	52 (50–55)
	NCJ	48	85 (76–93)	74 (64–84)	58 (50–66)
Energy^+ ^(150/6)	Gastric tube	216	79 (75–83)	95 (89–101)	78 (73–83)
(*n *= 359)	PEG	-	-	-	-
	Duodenal tube	143	81 (77–86)	102 (96–109)	82 (77–86)
	NCJ	-	-	-	-
Energy^+^/Protein^+ ^(204/7)	Gastric tube	155	79 (74–83)	82 (74–90)	59 (53–65)
(*n *= 80)	PEG	-	-	-	-
	Duodenal tube	99	81 (76–87)	85 (76–93)	60 (54–66)
	NCJ	14	90 (82–99)	70 (52–84)	91 (68–114)
Fiber^+ ^(106/4.1)	Gastric tube	47	71 (61–81)	67 (56–78)	52 (43–61)
(*n *= 69)	PEG	-	-	-	-
	Duodenal tube	22	71 (56–86)	47 (35–58)	37 (29–45)
	NCJ	-	-	-	-
Immunologically active (100/5.6)	Gastric tube	333	72 (69–76)	51 (46–55)	54 (50–58)
(*n *= 441)	PEG	1	-	-	-
	Duodenal tube	95	80 (74–86)	73 (63–83)	79 (70–89)
	NCJ	12	78 (59–97)	66 (48–83)	73 (54–92)
Semi-elemental (100/4)	Gastric tube	14	89 (73–105)	97 (68–126)	58 (42–74)
(*n *= 90)	PEG	-	-	-	-
	Duodenal tube	60	91 (86–96)	63 (54–171)	52 (46–57)
	NCJ	16	88 (69–106)	90 (65–116)	71 (51–90)
Standard/Energy^+ ^(125/5)	Gastric tube	350	82 (79–85)	85 (81–89)	68 (65–72)
(*n *= 539)	PEG	9	75 (50–101)	77 (52–102)	62 (42–81)
	Duodenal tube	168	85 (81–89)	90 (85–95)	74 (70–79)
	NCJ	12	89 (71–107)	138 (110–166)	110 (88–133)

Results are percentages of realized 'ideal' energy intake (30 kcal/kg body weight) and percentage of realized 'ideal' protein intake (1.5 g/kg) stratified by tube and by type of formula. CI, confidence interval; NCJ, needle catheter jejunostomy; PEG, percutaneous endoscopic gastrostomy.

**Table 3:** Days of successful intake divided by feeding route

Enteral route	Patients	Days of successful intake, %
Gastric tube	383	49 (1,188 of 2,424)
Duodenal tube [previous gastric tube]*	[116]	58 (564 of 979)
Needle catheter jejunostomy	17	76 (78 of 102)
Percutaneous endoscopic gastrostomy	3	57 (12 of 21)
Overall success of feeding intake	403	52 (1,842 of 3,526)

*All patients with a duodenal feeding tube were previously fed by gastric tube.

**Table 4:** Multivariate analysis of patient and feeding factors for the success of feeding intake

Feeding and patient factors	OR	95% CI
Duodenal tube*	1.44	1.21–1.73
Needle catheter jejunostomy*	3.32	2.05–5.38
Semi-elemental formula (100/4)^†^	3.02	1.75–5.21
Standard/Energy^+ ^formula (125/5)^†^	1.62	1.31–1.99
Mechanical ventilation	1.63	1.27–2.09
Contrast via tube to prepare for CT scan	0.34	0.22–0.55
Gastric retention (<200 ml/>200 ml)	1.51	1.29–1.78
Cisapride	0.83	0.71–0.96
TISS category per patient day	0.84	0.74–0.96
Elective admission	0.81	0.69–0.94
Length of stay in intensive care unit	1.53	1.28–1.82

Success of feeding intake was defined as feeding of more than 90% of the prescribed formula. CI, confidence interval; CT, computed tomography; OR, odds ratio; TISS, Therapeutic Intervention Score System. An odds ratio of more than 1 indicates improved success of feeding. *Compared with reference gastric tube (odds ratio of 1). ^†^Compared with the reference standard feeding (odds ratio of 1); numbers in parentheses are kcal/protein.

## References

[B1] Jolliet P, Pichard C, Biolo G, Chiolero R, Grimble G, Leverve X, Nitenberg G, Novak I, Planas M, Preiser JC (1998). Enteral nutrition in intensive care patients: a practical approach. Working Group on Nutrition and Metabolism, ESICM. European Society of Intensive Care Medicine. Intensive Care Med.

[B2] Kompan L, Kremzar B, Gadzijev E, Prosek M (1999). Effects of early enteral nutrition on intestinal permeability and the development of multiple organ failure after multiple injury. Intensive Care Med.

[B3] Marik PE, Zaloga GP (2001). Early enteral nutrition in acutely ill patients: a systematic review. Crit Care Med.

[B4] Minard G, Kudsk KA (1994). Is early feeding beneficial? How early is early?. New Horiz.

[B5] Perez J, Dellinger RP (2001). Other supportive therapies in sepsis. Intensive Care Med.

[B6] Jonkers CF, Prins F, Van Kempen A, Tepaske R, Sauerwein HP (2001). Towards implementation of optimum nutrition and better clinical nutrition support. Clin Nutr.

[B7] Cullen DJ, Civetta JM, Briggs BA, Ferrara LC (1974). Therapeutic intervention scoring system: a method for quantitative comparison of patient care. Crit Care Med.

[B8] Knaus WA, Draper EA, Wagner DP, Zimmerman JE (1985). APACHE II: a severity of disease classification system. Crit Care Med.

[B9] Stroud M, Duncan H, Nightingale J (2003). Guidelines for enteral feeding in adult hospital patients. Gut.

[B10] Shaw JH, Wildbore M, Wolfe RR (1987). Whole body protein kinetics in severely septic patients. The response to glucose infusion and total parenteral nutrition. Ann Surg.

[B11] Ishibashi N, Plank LD, Sando K, Hill GL (1998). Optimal protein requirements during the first 2 weeks after the onset of critical illness. Crit Care Med.

[B12] Heyland D, Cook DJ, Winder B, Brylowski L, Van deMark H, Guyatt G (1995). Enteral nutrition in the critically ill patient: a prospective survey. Crit Care Med.

[B13] Adam S, Batson S (1997). A study of problems associated with the delivery of enteral feed in critically ill patients in five ICUs in the UK. Intensive Care Med.

[B14] McClave SA, Sexton LK, Spain DA, Adams JL, Owens NA, Sullins MB, Blandford BS, Snider HL (1999). Enteral tube feeding in the intensive care unit: factors impeding adequate delivery. Crit Care Med.

[B15] De Jonghe B, Appere-De-Vechi C, Fournier M, Tran B, Merrer J, Melchior JC, Outin H (2001). A prospective survey of nutritional support practices in intensive care unit patients: what is prescribed? What is delivered?. Crit Care Med.

[B16] De B, Chapman M, Fraser R, Finnis M, De Keulenaer B, Liberalli D, Satanek M (2001). Enteral nutrition in the critically ill: a prospective survey in an Australian intensive care unit. Anaesth Intensive Care.

[B17] Heyland DK, Schroter-Noppe D, Drover JW, Jain M, Keefe L, Dhaliwal R, Day A (2003). Nutrition support in the critical care setting: current practice in canadian ICUs – opportunities for improvement?. JPEN J Parenter Enteral Nutr.

[B18] Parker D, Lawton R (2000). Judging the use of clinical protocols by fellow professionals. Soc Sci Med.

[B19] Krishnan JA, Parce PB, Martinez A, Diette GB, Brower RG (2003). Caloric intake in medical ICU patients: consistency of care with guidelines and relationship to clinical outcomes. Chest.

[B20] Meredith JW, Ditesheim JA, Zaloga GP (1990). Visceral protein levels in trauma patients are greater with peptide diet than with intact protein diet. J Trauma.

[B21] Ziegler F, Ollivier JM, Cynober L, Masini JP, Coudray-Lucas C, Levy E, Giboudeau J (1990). Efficiency of enteral nitrogen support in surgical patients: small peptides v non-degraded proteins. Gut.

[B22] Ritz MA, Fraser R, Tam W, Dent J (2000). Impacts and patterns of disturbed gastrointestinal function in critically ill patients. Am J Gastroenterol.

[B23] Montejo JC, Grau T, Acosta J, Ruiz-Santana S, Planas M, Garcia-De-Lorenzo A, Mesejo A, Cervera M, Sanchez-Alvarez C, Nunez-Ruiz R (2002). Multicenter, prospective, randomized, single-blind study comparing the efficacy and gastrointestinal complications of early jejunal feeding with early gastric feeding in critically ill patients. Crit Care Med.

[B24] Esparza J, Boivin MA, Hartshorne MF, Levy H (2001). Equal aspiration rates in gastrically and transpylorically fed critically ill patients. Intensive Care Med.

[B25] Heyland DK, Drover JW, MacDonald S, Novak F, Lam M (2001). Effect of postpyloric feeding on gastroesophageal regurgitation and pulmonary microaspiration: results of a randomized controlled trial. Crit Care Med.

[B26] Montecalvo MA, Steger KA, Farber HW, Smith BF, Dennis RC, Fitzpatrick GF, Pollack SD, Korsberg TZ, Birkett DH, Hirsch EF (1992). Nutritional outcome and pneumonia in critical care patients randomized to gastric versus jejunal tube feedings. The Critical Care Research Team. Crit Care Med.

[B27] Mallampalli A, McClave SA, Snider HL (2000). Defining tolerance to enteral feeding in the intensive care unit. Clin Nutr.

[B28] Spain DA, McClave SA, Sexton LK, Adams JL, Blanford BS, Sullins ME, Owens NA, Snider HL (1999). Infusion protocol improves delivery of enteral tube feeding in the critical care unit. JPEN J Parenter Enteral Nutr.

