# Supplementary material for: Daily enteral feeding practice on the ICU: attainment of goals and interfering factors
Source: Crit Care. 2005 Mar 22;9(3):R218–25. doi: 10.1186/cc3504 (PMC1175883; doi:10.1186/cc3504)
Supplement: Additional File 1 — A pdf file containing a table listing the protocol outline. [file cc3504-S1.pdf]

Additional file

Protocol outline: enteral nutrition in the ICU

Criterion	Action
Feeding schedule	Start 20 ml / hour<br>Increase 20 ml per day (day 1 to 5)<br>Optimal 100 ml per day
EN* in first 12 hour	GR† every 3 hours
GR < 200 ml	GR every 6 hours
GR < 200 ml	Return GR to the patient
GR > 200 ml	Discard GR, consult intensivist to start prokinetics
Prokinetics > 12 hours	GR > 200 reduce EN rate
Prokinetics and reduced EN rate and GR > 200 ml / 6 hours	Place duodenal tube
EN visible in month	Stop EN
Patient is vomiting	Stop EN
Symptoms of aspiration	Stop EN

* EN: Enteral Nutrition; † GR: Gastric Retention
